# Management of a Type I Hypersensitivity Reaction to IV Etoposide in a Woman with a Yolk Sac Tumor: A Case Report

**DOI:** 10.1155/2011/837160

**Published:** 2011-09-08

**Authors:** David Starks, Deborah Prinz, Amy Armstrong, Lindsay Means, Steven Waggoner, Robert DeBernardo

**Affiliations:** ^1^MacDonald Women's Hospital, University Hospitals Case Medical Center, Cleveland, OH 44106, USA; ^2^Women's Health Institute, The Cleveland Clinic Foundation, Cleveland, OH 44195, USA

## Abstract

Type I hypersensitivity reactions to intravenous administration of etoposide are extremely rare. Etoposide is an essential component of several chemotherapy regimens used in gynecologic oncology, and discontinuation of this drug during a course of treatment should only be due to severe patient intolerance. We report the successful use of intravenous etoposide phosphate as a substitute drug in a patient with a yolk sac tumor who manifested a Type I hypersensitivity to intravenous etoposide. The patient ultimately completed all 4 cycles of bleomycin, etoposide, cisplatin (BEP) using etoposide phosphate as a substitute drug.

## 1. Introduction

Hypersensitivity reaction to intravenous etoposide is a rare side effect and is infrequently reported in the medical literature. Etoposide is an essential component of chemotherapy for patients with rare gynecologic malignancies such as germ cell tumors and gestational trophoblastic disease, In young patients for whom etoposide-containing regimens are given with curative intent, omitting etoposide from regimens such as BEP (bleomycin, etoposide, cisplatin) or EMA-CO (etoposide, methotrexate, actinomycin D, cyclophosphamide, vincristine) may compromise efficacy, necessitating second-line therapy. There are no suitable substitutes for etoposide in these regimens that have demonstrably similar response rates. We report the first documented case in the gynecologic literature of a severe type I hypersensitivity reaction to etoposide in a woman being treated for a yolk sac tumor successfully treated with etoposide phosphate. 

## 2. Case

A 25 year old with stage IC yolk sac tumor was admitted to the hospital for her first cycle of BEP. She was premedicated with decadron, ondansetron, and acetaminophen. Within minutes of the etoposide infusion the patient reported shortness of breath, pruritus, and developed a rash on her face and chest. The patient became hypertensive and desaturated to 86% on pulse oximetry. The infusion was stopped after only 5 mL of etoposide had been administered. She was supplemented with oxygen and given diphenhydramine and decadron intravenously, which resolved her symptoms rapidly. The patient was rechallenged after administration of additional corticosteroids. The etoposide infusion was initiated at half the original rate and again the patient immediately developed shortness of breath, flushing, and hypoxia. The infusion was stopped and the patient's symptoms resolved. 

The following day the patient received an equivalent dose of IV etoposide phosphate, a water-soluble ester of etoposide after premedication with ranitidine (50 mg IV), diphenhydramine (50 mg IV), and hydrocortisone (100 mg IV). The patient tolerated the infusion well and did not exhibit any further hypersensitivity symptoms. The patient completed the prescribed four cycles of BEP using etoposide phosphate with the same regimen of premedication without incident, and is currently free of disease. 

## 3. Discussion

A type I hypersensitivity reaction to the IV administration of etoposide is an unusual event impacting less than 1% of patients. The etiology is unknown, although it is speculated that patients are reacting to the polysorbate 80 used to dissolve etoposide. There have been no documented cases of a hypersensitivity reaction to the administration of oral etoposide, and in animal models, reaction to polysorbate have been demonstrated to result in the release of histamine [1]. Etoposide phosphate is a water soluble prodrug of etoposide, and this variant does not require polysorbate 80 to enter solution. To date no hypersensitivity reaction to etoposide phosphate has been documented [2]. There are few case reports in the literature supporting the use of etoposide phosphate in patients with a hypersensitivity reaction to etoposide [3, 4]. This is the first case report in the gynecologic literature of a patient with a yolk sac tumor and a type I hypersensitivity reaction to etoposide successfully treated with 4 cycles of IV etoposide phosphate. 

Although infrequently used by gynecologic oncologists, etoposide is an important agent in treating rare and aggressive gynecologic malignancies. Eliminating etoposide in this regimen would likely negatively impact this patient's potential for a cure. Alternative strategies included desensitization: increasing the dose of steroid premedication and slowing the rate of infusion, but these strategies failed in our patient. Consideration was given to substituting oral etoposide. However, there is no data in the gynecologic or testicular cancer literature to support this and may have compromised her chance of cure. The use of second-line chemotherapy such as VAC (vincristine, adriamycin, cyclophosphamide) was also considered but given the lower response rate this strategy was abandoned. The use of etoposide phosphate in this setting seemed wise. 

We were able to successfully complete four cycles of BEP using etoposide phosphate, a water soluble prodrug form of etoposide. We initially premedicated our patient with H1 and H2 blockers and 100 mg of IV hydrocortisone 15 minutes before and repeated the hydrocortisone dose immediately following infusion. After successfully completing the first cycle of therapy, we premedicated with diphenhydramine, ranitidine, and hydrocortisone prior to infusing the etoposide phosphate. The patient tolerated treatment without further incident. 

Based on our experience with etoposide phosphate in this patient, we have created a protocol for its use in the setting of hypersensitivity reactions. While an extremely rare side effect to the infusion of etoposide, hypersensitivity reactions present a therapeutic challenge with few viable alternatives. Etoposide phosphate, however, appears to be a suitable alternative and should be considered the alternative drug of choice for patients with a hypersensitivity reaction to IV etoposide. 
